# Targeting CDK9 for treatment of colorectal cancer

**DOI:** 10.1002/1878-0261.12559

**Published:** 2019-08-21

**Authors:** Muhammed H. Rahaman, Frankie Lam, Longjin Zhong, Theodosia Teo, Julian Adams, Mingfeng Yu, Robert W. Milne, Chris Pepper, Noor A. Lokman, Carmela Ricciardelli, Martin K. Oehler, Shudong Wang

**Affiliations:** ^1^ Centre for Drug Discovery and Development School of Pharmacy and Medical Sciences University of South Australia Cancer Research Institute Adelaide SA Australia; ^2^ School of Medicine Cardiff University, Health Park UK; ^3^ Discipline of Obstetrics and Gynaecology Adelaide Medical School University of Adelaide SA Australia

**Keywords:** anti‐proliferation, apoptosis, cancer therapy, CDK9, colorectal cancer, RNAPII transcription

## Abstract

Colorectal cancer (CRC) remains one of the most lethal human malignancies, and pursuit of new therapeutic targets for treatment has been a major research focus. Cyclin‐dependent kinase 9 (CDK9), which plays a crucial role in transcription, has emerged as a target for cancer treatment. CDKI‐73, one of the most potent and pharmacologically superior CDK9 inhibitors, has demonstrated excellent anti‐tumour efficacy against several types of cancers. In this study, we evaluated its therapeutic potential against CRC. CDKI‐73 elicited high cytotoxicity against all colon cancer cell lines tested. Cell cycle and apoptosis analysis in HCT 116 and HT29 cells revealed that CDKI‐73 induced cell death without accumulation of DNA at any phase of the cell cycle. Moreover, it caused depolarisation of mitochondrial membrane, leading to caspase‐independent apoptosis. Knockdown by shRNA demonstrated the CDK9‐targeted mechanism of CDKI‐73, which also affected the Mnk/eIF4E signalling axis. In addition, RT‐qPCR analysis showed that CDKI‐73 down‐regulated multiple pro‐survival factors at the mRNA level. Its *in vivo* anti‐tumour efficacy was further evaluated in Balb/c nude mice bearing HCT 116 xenograft tumours. CDKI‐73 significantly inhibited tumour growth (***P <* *0.001) without overt toxicity. Analysis of the tumour tissues collected from the xenografted animals confirmed that the *in vivo* anti‐tumour efficacy was associated with CDK9 targeting of CDKI‐73. Overall, this study provides compelling evidence that CDKI‐73 is a promising drug candidate for treating colorectal cancer.

**Abbreviations**
4E‐BP: eIF4E‐binding protein
CDK: cyclin‐dependent kinase
CRC: colorectal cancer
eIF4E: eukaryotic translation initiation factor 4E
Erk: extracellular signal‐regulated kinase
HCCLs: human colon cancer cell lines
IC_50_: half‐maximal inhibitory concentration
IHC: immunohistochemisty
IP: intraperitoneal injection
JC‐1: 5,5′,6,6′‐tetrachloro‐1,1′3,3′‐tetraethylbenzimidazolylcarbocyanine iodide
KD: knockdown
MAPK: mitogen‐activated protein kinase
MMP: mitochondrial membrane potential
Mnk: mitogen‐activated protein kinase‐interacting kinase
mTOR: mammalian target of rapamycin
MTT: 3‐(4,5‐dimethylthiazol‐2‐yl)‐2,5‐diphenyltetrazolium bromide
PARP: poly (ADP‐ribose) polymerase
PI: propidium iodide
PO: oral dosing
p‐RNAPII^Ser2^: phosphorylated RNAPII serine 2
Q3D: once every 3 days dosing
Q7D: once‐a‐week dosing
RNAPII: RNA polymerase II
rpS6: ribosomal protein S6
RT‐qPCR: real‐time quantitative polymerase chain reaction
shRNA: short hairpin RNA
XIAP: X‐linked inhibitor of apoptosis

## 1 Introduction

Colorectal cancer (CRC) is one of the most lethal malignant neoplasms worldwide. It shows substantial genetic heterogeneity and aberration in multiple cellular pathways among the subtypes (Hahn *et al*., 2016). Clinical, morphological and molecular features of CRC assisted to create a defined classification of the disease subtypes, facilitating the prospect of personalised treatment for CRC patients. Despite the availability of defined classification of the disease subtypes and immense improvement in systemic therapy, not all the subtypes are susceptible to currently available treatments. Moreover, in the metastatic incurable form of CRC, treatment with cytotoxic chemotherapy is able to achieve 5‐year survival rate only in 12.5% of the patients (Siegel *et al*., 2014). On the other hand, targeted therapies such as cetuximab or panitumumab as monotherapy have very low response rates of 10–20% and ultimately progression of disease occurs due to resistance (Bardelli and Siena, 2010; Cunningham *et al*., 2004). Development of resistance to cytotoxic (e.g. Fluoropyrimidines) and targeted therapies (e.g. EGFR‐targeted therapies cetuximab or panitumumab) remains the primary reason for treatment failure. Thus, development of new therapeutic targets that can translate into better clinical outcomes is urgently needed for CRC patients.

Cyclin‐dependent kinase 9 (CDK9) is such a potential target for the treatment of CRC which plays a crucial role in the elongation step of global transcription process. Multiple studies using small molecule inhibitors and RNA interference have demonstrated that oncogenic transcription driven by CDK9 increases the survival and proliferation of cancer cells (Chen *et al*., 2005; Huang *et al*., 2014) and hence is continuously targeted for cancer treatment (Baker *et al*., 2016; Morales and Giordano, 2016; Rahaman *et al*., 2016). As CDK9 plays such a critical role in the transcription of a number of oncogenes (Krystof *et al*., 2012; Lam *et al*., 2001; Wang and Fischer, 2008), targeting CDK9 may provide a promising therapeutic strategy for the treatment of CRC.

CDKI‐73 is a highly potent small molecule inhibitor of CDK9 (Lam *et al*., 2014; Shao *et al*., 2013). It demonstrated potent cytotoxic effects on a wide range of cancer cell lines from different origins, but has proved to be less toxic towards normal cells (Lam *et al*., 2014; Walsby *et al*., 2014). CDKI‐73 also demonstrated a synergistic effect with fludarabine through a mechanism associated with the transcriptional inhibition of multiple anti‐apoptotic proteins such as Mcl‐1, Bcl‐2 and XIAP, which represented a promising therapeutic strategy for treating CLL‐ and fludarabine‐relapsed diseases. Moreover, recent knockdown studies in A2780 ovarian cancer cells revealed that the down‐regulation of CDK9 affected the oncogenic translation via the Mnk‐eIF4E axis (Lam *et al*., 2014).

Among several CDK9 inhibitors in the clinical trials, flavopiridol (Alvocidib) is the first generation pan‐CDK inhibitor with highest potency against CDK9 (Bose *et al*., 2013). Flavopiridol has significant activity in patients with relapsed or refractory chronic lymphocytic leukaemia and has received orphan drug designation for treatment of CLL from the U.S. FDA. However, its clinical development as therapy has been hampered by a high incidence of serious toxicity necessitating inpatient treatment. Nevertheless, its low dose in combination with chemotherapy agents has been found to be effective in a phase II trial of patients with advanced colorectal cancer (Aklilu *et al*., 2003). Dinaciclib is the most advanced CDK9 inhibitor currently in phase III clinical trials against CLL as a single agent and in phase II trials against different haematological malignancies as well as some solid tumours. It also inhibits multiple CDK, most notably CDK1, CDK2, CDK5 and CDK9 with half‐maximal inhibitory concentration (IC_50_) values of 3, 1, 1 and 4 nm, respectively (Parry *et al*., 2010), which exhibited superior activity. A phase I dose escalation study of dinaciclib in advanced malignancies with a majority of colorectal cancer patients showed disease stabilisation and had better safety profile compared with flavopiridol. However, both of these inhibitors suffer from lack of prolonged CDK9 inhibition due to poor pharmacokinetic properties (Flinn *et al*., 2005; Gojo *et al*., 2013). Thus, new inhibitors with improved pharmacological properties are in high demand.

The current study explored the therapeutic potential of CDKI‐73, an oral and highly potent CDK9 inhibitor, by examining its effect on human colon cancer cell lines (HCCL) of different clinical relevance. CDKI‐73 effectively induced apoptosis through the inhibition of CDK9 activity which also targeted Mnk‐eIF4E signalling as a downstream effect of CDK9 inhibition. A preclinical xenograft model of HCT 116 human colorectal tumour validated the efficacy of CDKI‐73 and its CDK9 targeting effect in the tumour was confirmed. Based on the findings, CDK9 inhibition represents a promising therapeutic approach and CDKI‐73 is a promising drug candidate for treating colorectal cancer.

## 2 Materials and methods

### 2.1 Chemical compounds

CDKI‐73 was synthesized (Shao *et al*., 2013) and kindly provided by Changzhou LeSun Pharmaceuticals Ltd., Changzhou, China. Doxorubicin and flavopiridol were purchased from Sigma‐Aldrich (Castle Hill, NSW, Australia). Z‐VAD‐FMK was purchased from Merck (Kilsyth, Vic., Australia). All the compounds were dissolved in dimethylsulphoxide (DMSO) at a concentration of 10 mm stock and stored at −20 °C.

### 2.2 Cell culture

All HCCL were obtained from the cell bank at the Centre for Drug Discovery and Development, University of South Australia. Cells were cultured in Roswell Park Memorial Institute (RPMI)‐1640 medium supplemented with 10% FBS at 37 °C in a 5% CO_2_ atmosphere.

### 2.3 Cell viability and caspase‐3/7 activity assays

To estimate the viability of all HCCL as reported previously, 3‐(4,5‐dimethylthiazol‐2‐yl)‐2,5‐diphenyltetrazolium bromide (MTT; Sigma‐Aldrich) was used (Wang *et al*., 2004). Concentrations of the compounds required to reduce cell viability by 50% (IC_50_) were calculated using graphpad prism 7.02 (La Jolla, CA, USA). Caspase‐3/7 activity was measured using an Apo‐ONE homogeneous caspase‐3/7 kit (Promega, Madison, WI, USA) according to the manufacturers’ instructMitoions and analysed using an EnVision multi‐label plate reader (PerkinElmer, Beaconsfield, UK).

### 2.4 Cell cycle analysis, detection of apoptosis and mitochondrial membrane potential

For cell cycle analysis in HCT 116 and HT29 cells, 8 × 10^4^ cells were seeded and incubated overnight at 37 °C in a 5% CO_2_ atmosphere. Following exposure to each compound for 24 h, cells were trypsinised and centrifuged (300 ***g***, 5 min) to collect the cell pellets, which were then fixed with 70% ethanol on ice for 15 min. They were then centrifuged again to collect the pellets and incubated with staining solution [50 μg·mL^−1^ propidium iodide (PI), 0.1 mg·mL^−1^ ribonuclease A, 0.05% Triton X‐100] at 37 °C for 1 h, followed by analysis using CytoFLEX flow cytometer (Beckman Coulter, Brea, CA, USA). For the detection of apoptosis and cellular mitochondrial membrane potential (MMP) in HCT 116 cells, annexin V/PI double‐staining and JC‐1 staining methods were followed, respectively. Commercially available apoptosis detection kit and MitoScreen (JC‐1) kit were purchased from Becton Dickinson, North Ryde, NSW, Australia. Following the manufacturers' instructions, samples were analysed using the CytoFLEX or Gallios flow cytometer (Beckman Coulter) within 1 h of staining. Data were analysed using cytexpert 2.1 or kaluza v1.2 software (Beckman Coulter).

### 2.5 CDK9 knockdown in HCT 116 and HT29 cells

For stable knockdown in HCT 116 and HT29 cells, MISSION shRNA clones targeting human CDK9 (shCDK9#1: TRCN0000199892, shCDK9#2: TRCN0000000494 and shCDK9#3: TRCN0000000497), a pLKO.1‐puro empty vector and a non‐targeted shRNA were used. Plasmids containing shRNA clones and controls were purchased from Sigma‐Aldrich. Following the manufacturer's instructions, the cells were transfected with the CDK9 shRNA and control plasmids using Lipofectamine 3000 Reagent (Life Technologies, ​Mulgrave, Vic., Australia). Transfected cells were selected by adding puromycin (1 μg·mL^−1^) to the culture for 2 weeks. The relative expression of CDK9 was assessed subsequently by real‐time quantitative PCR (RT‐qPCR) and western blotting.

### 2.6 Western blotting

Western blotting with cell lysates was performed either following Simple Western™ assays (ProteinSimple, Santa Clara, CA, USA) according to the manufacturers’ instructions (Rustandi *et al*., 2012) or as previously described (Liu *et al*., 2012). For xenograft tumour tissues, western blot was performed following a modified method described previously (Booher *et al*., 2014). Briefly, the lysates were prepared by sonicating (Qsonica Sonicator, Melville, NY, USA) the smashed tumour tissues in a 2.5 μL·mg^−1^ lysis buffer (containing protease and phosphatase cocktail inhibitors). Supernatants were collected after centrifuging for 15 min at 20 000 ***g*** at 4 °C. Antibodies used were as follows: total RNAPII, phosphorylated RNAPII serine 2 (p‐RNAPII^Ser2^) and serine 5 (p‐RNAPII^Ser5^) (Covance, Princeton, NJ, USA), 4E‐BP1, p‐4E‐BP1^Thr70^, β‐actin, procaspase‐3, procaspase‐7, CDK9, c‐Myc, eIF4E, p‐eIF4E^Ser209^, eIF4G, p‐Erk^Thr202/Tyr204^, p‐p38^Thr180/Tyr182^, p38, rpS6, Mcl‐1, Mnk1, PARP, cleaved PARP (Cell Signaling Technology, Danvers, MA, USA), Erk (ProteinSimple or Cell Signaling Technology), MDM‐2 (Becton Dickinson), Bcl‐2, cyclin D1, p‐S6^Ser240/244^, and p53 (Dako, Glostrup, Denmark). Both anti‐mouse and anti‐rabbit immunoglobulin G horseradish peroxidase‐conjugated antibodies were obtained from Dako.

### 2.7 Real‐time quantitative PCR

RNA extraction was performed using the High Pure RNA Isolation Kit (Roche Applied Science, Castle Hill, NSW, Australia). Using the Transcriptor First Strand cDNA Synthesis Kit (Roche Applied Science, Castle Hill, Australia), 1 μg of RNA was used in a 20‐ μL reverse transcription reaction. RT‐qPCR was carried out in duplicate with cDNA using SYBR Green I dye (Roche Applied Science, Castle Hill, Australia) and performed using a LightCycler^®^ 96 instrument (Roche Applied Science, Penzberg, Germany). Relative quantification using E‐method established by Roche Applied Science (Tellmann, 2006) was performed with β‐Actin as reference gene and untreated samples as study calibrators. The sequences of primers and amplification efficiency (*E*) were as follows: β‐actin: 5′‐ACTCTTCCAGCCTTCCTTC‐3′ (forward) and 5′‐GATGTCCACGTCACACTTC‐3′ (reverse), *E* = 1.72; BCL‐2: 5′‐ATGGGATCGTTGCCTTATGC‐3′ (forward) and 5′‐CAGTCTACTTCCTCTGTGATGTTGT‐3′ (reverse), *E* = 1.89; CDK9: 5′‐AAAACGAGAAGGAGGGGTTCC‐3′ (forward) and 5′‐CCTTGCAGCGGTTATAGGGG‐3′ (reverse), *E* = 1.92; CYCLIN D1: 5′‐TTTCTGATGAAATCAGCCCTAGT‐3′ (forward) and 5′‐GCACGGTCAGGTGACACATA‐3′ (reverse); *E* = 1.72; MCL‐1: 5′‐AACAAAGAGGCTGGGATGGG‐3′ (forward) and 5′‐TGCCAAACCAGCTCCTACTC‐3′ (reverse); *E* = 1.80; MNK1: 5′‐AAGGCCATTGAGACACTTCG‐3′(forward) and 5′‐CCCAAATGAAATAAAGCTCCTG‐3′ (reverse); 4E‐BP1: 5′‐TGACCAAAACACCCCCAAGG‐3′ (forward) and 5′‐TGTGACTCTTCACCGCCCG‐3′ (reverse).

### 2.8 *In vivo* studies

The *in vivo* studies were conducted following the approved protocol from the institutional animal ethics committee, and approval for the *in vivo* xenograft study (project number: U15‐14) was provided by the University of South Australia animal ethics committee (Adelaide, Australia). An HCT 116 xenograft model was established as described previously (Lu *et al*., 2014). Briefly, female nude (nu/nu) Balb/c mice at 6–8 weeks were inoculated subcutaneously on their hind flanks with 2.5 × 10^6^ HCT 116 cells suspended in RPMI medium. Tumour‐bearing mice were randomly allocated to different treatment groups (*n* = 8 mice per group) and the treatments were commenced when the average tumour volume reached ~ 100 mm^3^. CDKI‐73 or vehicle control (5% *N*‐methyl‐2‐pyrrolidone/95% polyethylene glycol‐400) was administered by oral gavage once every 3 days (Q3D). Cisplatin, the positive control, was dissolved in 0.9% normal saline and administered by intraperitoneal injection once per week (Q7D). Mice were humanely killed if they reached the clinical endpoint defined by the approved protocol (tumour volume ≥ 2000 mm^3^ or body weight loss ≥ 15% of the pre‐treatment weight). Tumours from five mice from each group (vehicle and 100 mg·kg^−1^ CDKI‐73) were collected for western blot analysis and immunohistochemistry (IHC).

### 2.9 Immunohistochemistry

Immunohistochemistry with the tumour tissues collected from the xenograft study was performed as previously described (Lokman *et al*., 2013). Briefly, the formalin‐fixed tumour tissues were processed, embedded in paraffin and then sectioned (5 μm). The tissues then underwent antigen retrieval (5 min at 750 W, 15 min at 350 W in a microwave) in 10 mm citrate buffer (pH 6.5). The tissues were immunostained with Ki67 (rabbit monoclonal, 1/400; Epitomics, Burlingame, CA, USA) as described previously (Ricciardelli *et al*., 2011). Streptavidin‐peroxidase conjugate (1/500; Dako) and diaminobenzidine tetrahydrochloride (DAB; Sigma‐Aldrich) were used to visualise the staining. Images were recorded using a NanoZoomer Digital Pathology System (Hamamatsu Photonics, SZK, Japan).

### 2.10 Statistical analysis

All the *in vitro* data are presented as mean ± standard deviation (SD) and representative figures are provided. Representative graphs or figures are presented from at least three independent experiments. In the *in vivo* study, the data are presented as mean ± standard error of mean (SEM). The statistically significant differences between the groups were analysed by appropriate unpaired *t*‐test or one‐way ANOVA. All statistical analyses were performed using graphpad prism 7.03. The significance level was set as *P ≤ *0.05.

## 3 Results

### 3.1 CDKI‐73 potently inhibits the growth and activates caspase‐3/7 in HCCL

**Figure 1:**
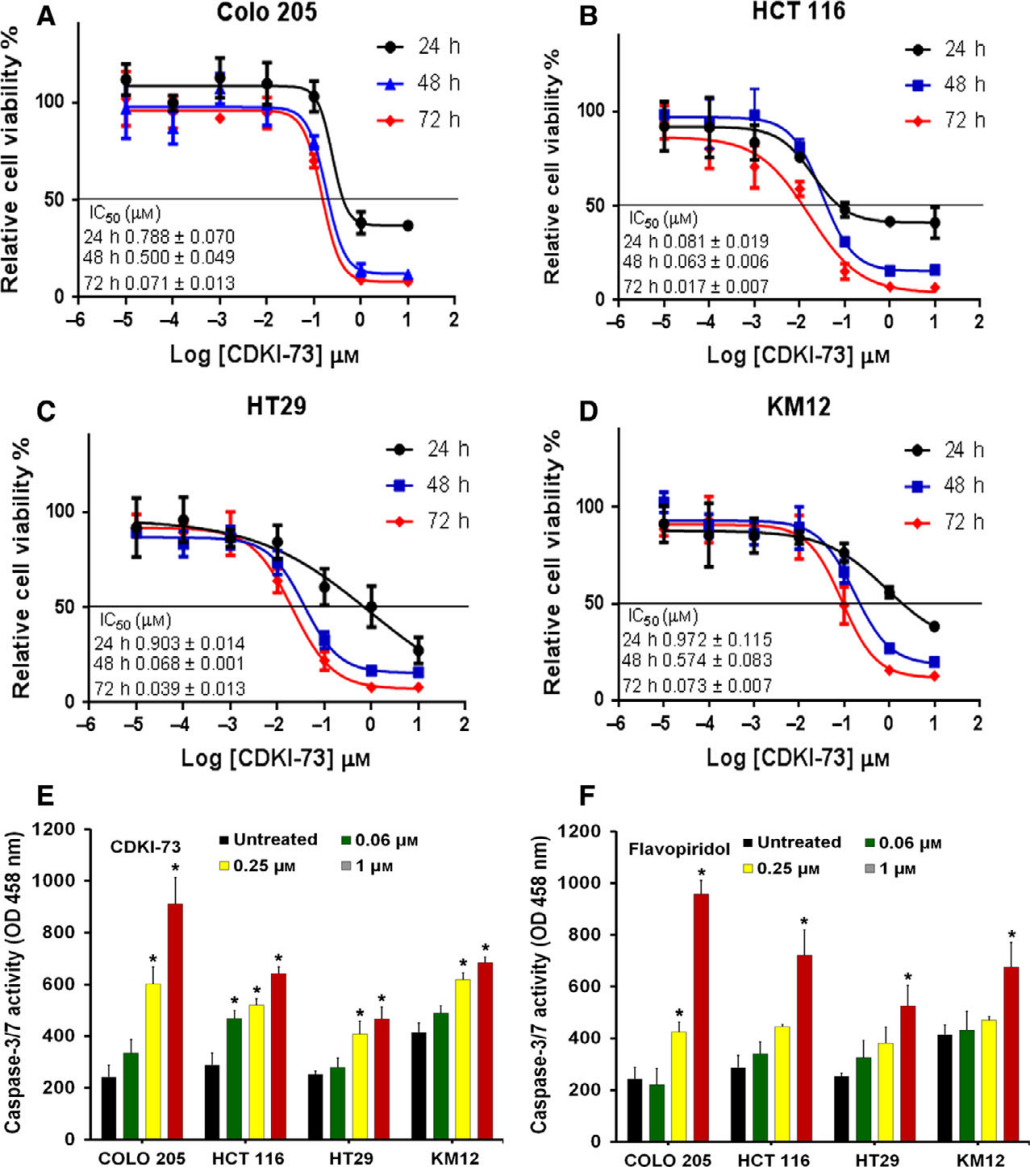
Growth inhibitory profile of CDKI‐73 against HCCL. (A–D) Representative dose response curves for the Colo 205, HCT 116, HT29 and KM12 cells after treatment with CDKI‐73 for indicated time points. IC_50_ values are shown. Caspase‐3/7 activity in HCCL following the treatment with (E) CDKI‐73 or (F) flavopiridol for 24 h. Data represent the mean ± SD of at least three independent experiments. *Statistical significance (*P *≤* *0.05) compared with untreated control determined using unpaired *t‐*test.

CDKI‐73 was identified through structure‐guided medicinal chemistry and is one of the most potent among existing CDK9 inhibitors (Shao *et al*., 2013; Walsby *et al*., 2014). It exhibited marked anti‐cancer activity against a wide range of cancer cell lines (Lam *et al*., 2014). To determine the cellular potency of CDKI‐73 and its effect on the viability of HCCL, we use a panel of four well‐characterised HCCL (COLO 205, HCT 116, HT29 and KM12) representing different stages of the disease (Huang *et al*., 2000; Schneider *et al*., 2012; Svingen *et al*., 2004). CDKI‐73 exhibited anti‐proliferative activities against all the cell lines in a dose‐ and time‐dependent manner, with IC_50_ values ranging from 0.017 to 0.972 μm, being most potent against HCT 116 (Fig. 1A–D). CDKI‐73 potently inhibited the growth of HCT 116 cells as early as 24 h, with an IC_50_ value of 0.081 μm (flavopiridol, IC_50_ = 0.068 μm) (Fig. 1B, Table S1). Prolonged exposure (i.e. 48 and 72 h) to CDKI‐73 caused further reduction in cell viability.

To investigate the apoptotic mechanism, the activity of caspase‐3/7 was measured in each HCCL after 24‐h exposure to CDKI‐73. Flavopiridol was used as a comparator. As shown in Fig. 1E, CDKI‐73 caused a dose‐dependent activation of caspase‐3/7 starting from 0.06 μm concentration, and significant levels (*P *<* *0.05) of caspase activation were observed at 0.25 and 1 μm concentrations. The different concentrations of CDKI‐73 required for activation of caspase3/7 in the HCCL were consistent with the anti‐proliferative activities by 24‐h MTT assays (Fig. 1A–D). Flavopiridol exhibited similar profiles in caspase‐3/7 activation on these HCCL to CDKI‐73 (Fig. 1F).

### 3.2 CDKI‐73 induces apoptosis without affecting cell cycle

**Figure 2:**
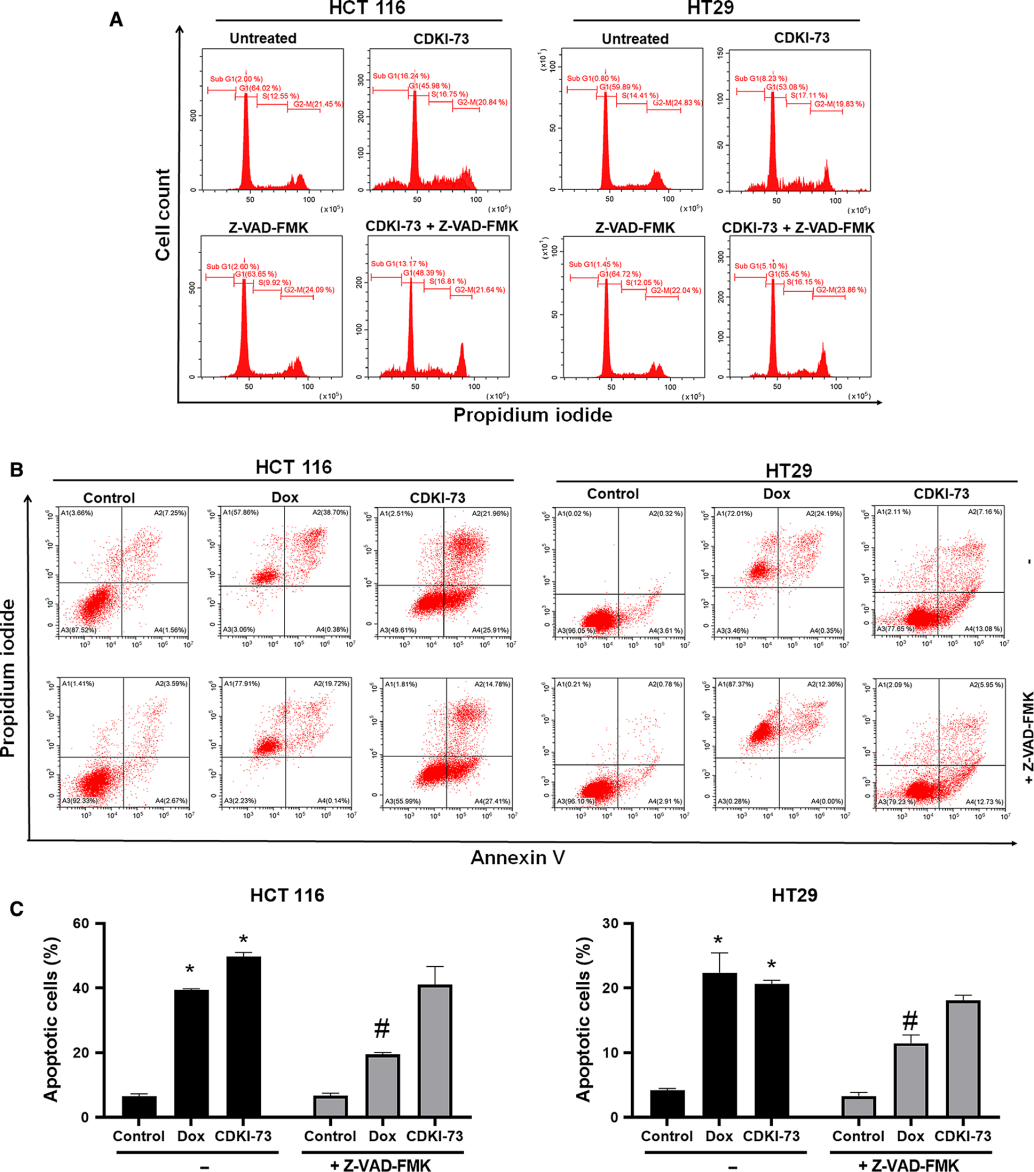
Induction of apoptosis by CDKI‐73 in HCT 116 and HT29 cells. (A) Cell cycle analysis of HCT 116 and HT29 cells after exposure to 0.25 μm CDKI‐73 for 24 h. (B) Apoptosis analysis of HCT 116 and HT29 cells after incubation with CDKI‐73 for 48 h by annexin V/PI assay. Co‐treatment of CDKI‐73 with Z‐VAD‐FMK (20 μm) was performed to demonstrate the caspase independency. Representative cytometric plots are presented. (C) Quantification of apoptotic cells. Untreated cells were used as negative control. Doxorubicin (Dox, 1 μm) was used as positive control. Data presented as mean ± SEM of three independent experiments. For each treatment condition, 10 000 events were recorded and cellular debris was excluded from the analysis. *P <* *0.05 compared with negative control; ^#^ *P *<* *0.05 compared with respective treatment without Z‐VAD‐FMK determined by ANOVA followed by Student–Newman–Keuls test.

Our previous studies showed that CDKI‐73 also inhibits CDK1 and CDK2 in kinase assays (Walsby *et al*., 2014). Both CDKs have a profound effect on the DNA contents of the cells in the respective G1‐S and G2‐M phase of the cell cycle. Thus, we investigated whether CDKI‐73 and knockdown of CDK9 (CDK9KD) affected the cell cycle distribution of HCT 116 and HT29 cells. Figure 2A shows no detectable accumulation of cells in any particular phase of the cycle in HCT 116 and HT29 cells treated with CDKI‐73 (0.25 μm). Similarly, no significant cell cycle arrest was observed in CDK9KD cells except accumulation of cells in sub‐G1 phase. There was no significant difference between the transfection controls (i.e. empty vector and non‐target shRNA) and untransfected cells (Figs 2A and S1). A substantial increase in the sub‐G1 population of the both CDKI‐73‐treated and CDK9KD cells indicated the occurrence of cell death. HCT 116 and HT29 cells were treated with either a pan‐caspase inhibitor Z‐VAD‐FMK (20 μm) or Z‐VAD‐FMK plus CDKI‐73. There were no noticeable changes in the percentage of the sub‐G1 population, suggesting that CDKI‐73 can induce cell death in a caspase‐independent manner, as further confirmed by apoptosis analysis.

Further apoptosis analysis utilising annexin V/PI double‐staining assay revealed that 0.25 μm CDKI‐73 induced apoptosis (annexin V^+^/PI^−^ and annexin V^+^/PI^+^) in ~ 50% of HCT 116 and ~ 20% of HT29 cells. Co‐treatment of CDKI‐73 with Z‐VAD‐FMK resulted in a similar trend of apoptotic effect (~ 39% in HCT 116 and ~ 18% in HT29 cells) when compared with the treatment with CDKI‐73 alone (Fig. 2B,C). Taken together, CDKI‐73 induced both caspase‐dependent and ‐independent apoptosis in HCT 116 and HT29 cells. The presence of caspases increased the susceptibility (i.e. ~ 10% increased apoptosis in HCT 116 cells) of cell death driven by the pharmacologic inhibition. Doxorubicin (1 μm) was used as positive control for this experiment. Co‐treatment with Z‐VAD‐FMK significantly reduced the doxorubicin‐induced apoptosis in both HCT 116 (from ~ 40% to ~ 20%) and HT29 (from ~ 25% to ~ 12%) cells.

### 3.3 CDK9 inhibition leads to the loss of mitochondrial integrity in cancer cells

**Figure 3:**
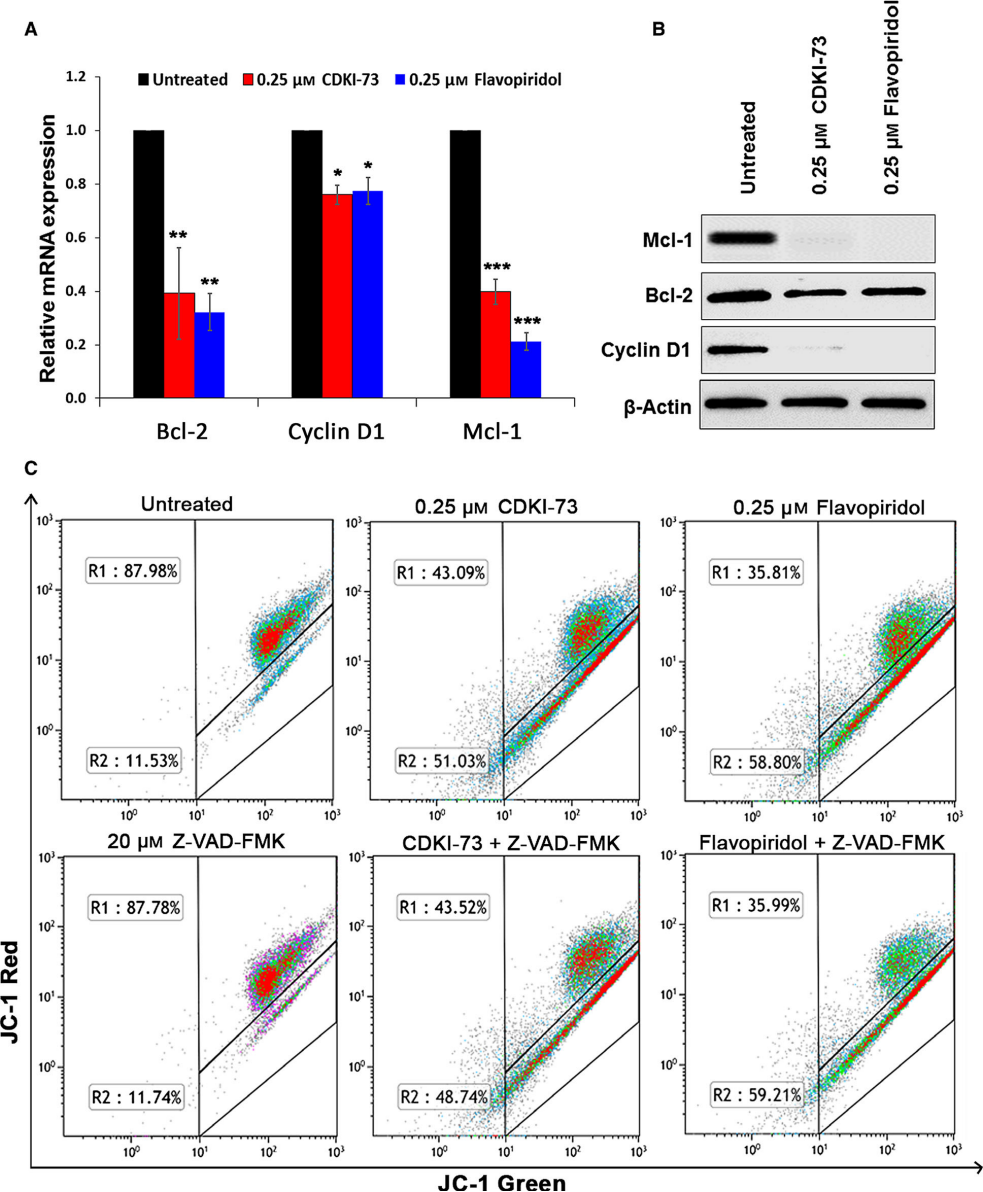
Inhibition of CDK9 reduced mitochondrial membrane potential. (A) RT‐qPCR showed relative mRNA levels of Bcl‐2, cyclin D1 and Mcl‐1 in HCT 116 cells after exposure to CDKI‐73 or flavopiridol for 4 h, normalised against β‐actin. Data presented as mean ± SD of three independent experiments; *P ≤* *0.05, **P ≤* *0.01, ***P ≤* *0.001 (unpaired *t*‐test). (B) Western blot analysis of the cells after treatment with CDKI‐73 or flavopiridol for 24 h. **(**C**)** Cellular MMP was determined by JC‐1 assay in HCT 116 cells after 48‐h treatment with CDKI‐73 or flavopiridol. Co‐treatment with Z‐VAD‐FMK (20 μm) was performed to demonstrate the caspase independency.

CDK9 activity in cardiac tissue has been known for its transcriptional suppression of genes required for normal mitochondrial function, such as PGC‐1α and its downstream effectors, resulting in cardiac hypertrophy (Sano *et al*., 2004; Ventura‐Clapier *et al*., 2008). However, in cancer, the role of CDK9 in mitochondrial function has not been elucidated. CDK9 regulates the expression of anti‐apoptotic proteins such as Bcl‐2, cyclin D1 and Mcl‐1, which play roles in mitochondrial function (Hardwick and Soane, 2013; Perciavalle *et al*., 2012; Sakamaki *et al*., 2006). Using RT‐qPCR, we showed that transcription of these genes was reduced in HCT 116 cells after 4 h of exposure to 0.25 μm CDKI‐73 or flavopiridol (Fig. 3A). Western blotting confirmed that the corresponding protein levels were also reduced (Fig. 3B). Loss of mitochondrial membrane potential leading to passage of ions across mitochondria and release of cytochrome *c* into cytoplasm is a distinctive feature of programmed cell death at early stage. The effect of CDKI‐73 on the mitochondrial membrane potential (MMP) of HCT 116 cells was assessed by JC‐1 assay, which determines the polarity of cellular mitochondria. After 48 h of exposure to 0.25 μm CDKI‐73 or flavopiridol, the level of MMP in HCT 116 cells was reduced in a caspase‐independent manner (Fig. 3C). Depolarisation of cellular mitochondria, initiated through transcriptional inhibition by CDKI‐73, presented the cells with mitochondria‐dependent apoptosis as an alternative mechanism for cell death.

### 3.4 CDKI‐73 targets CDK9‐mediated transcription leading to down‐regulation of eIF4E‐mediated translation

**Figure 4:**
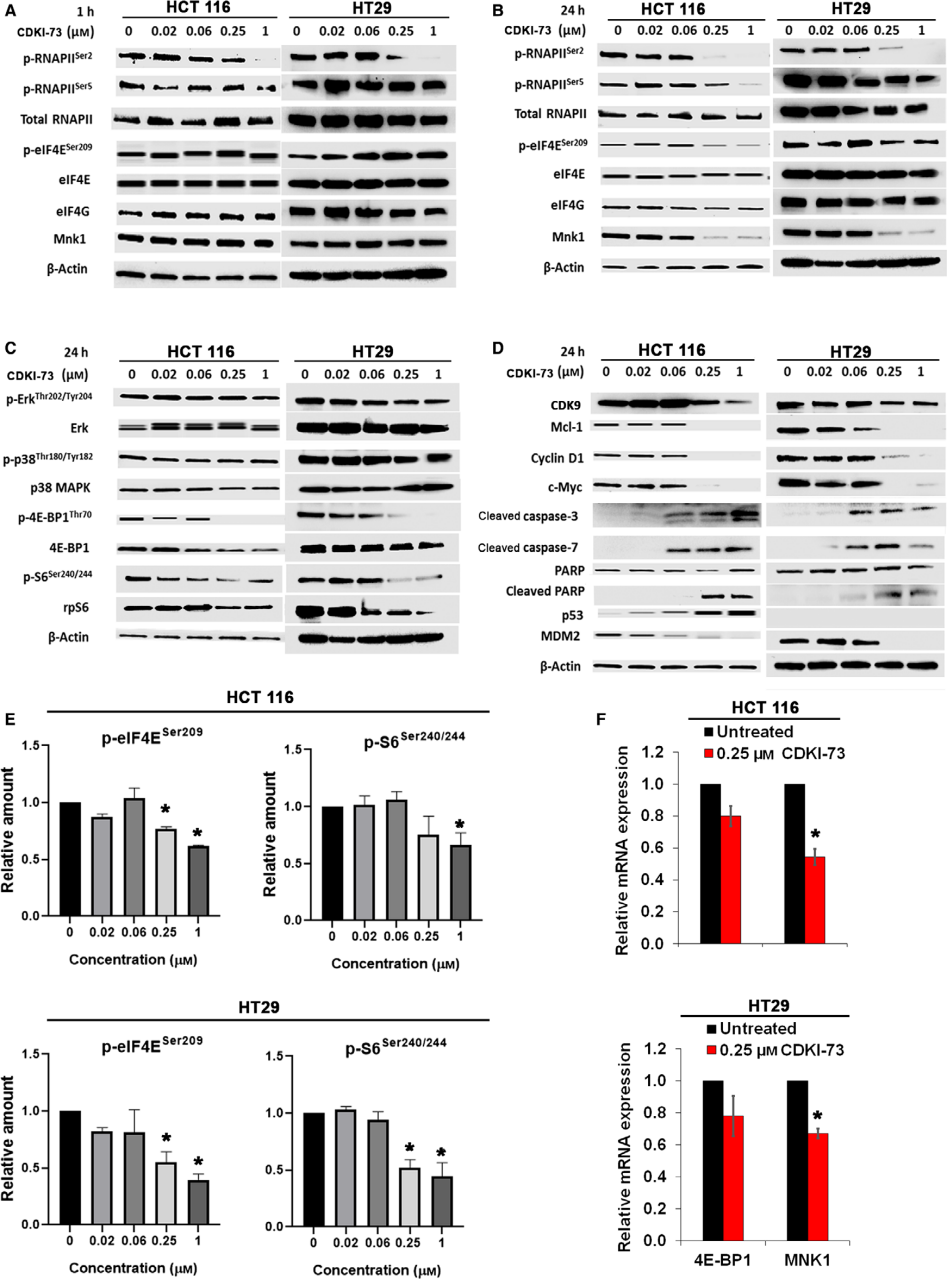
Cellular mode of action of CDKI‐73. (A) Western blot analysis after 1‐h treatment of HCT 116 and HT29 cells with CDKI‐73 at different concentrations. (B–D) Western blot analysis showing downstream effects of CDKI‐73 on Mnk/eIF4E‐mediated translational pathway in HCT 116 and HT29 cells after 24 h treatment. β‐Actin was used as an internal loading control. (E) Bar graph showing relative amount of p‐eIF4E^Ser209^ and p‐S6^Ser240/244^ in HCT 116 (top) and HT29 (bottom) (normalised to loading control, mean ± SD, *n* = 3). (F) RT‐qPCR showing the relative mRNA levels of MNK1 and 4E‐BP1 after 4‐h treatment with 0.25 μm CDKI‐73. β‐Actin was used as a reference gene. *P ≤* *0.05 (unpaired *t*‐test).

Western blot analysis was performed to study the biological response of CDK9 inhibition by CDKI‐73 and its mechanism of action in HCT 116 and HT29 cells. CDKI‐73 potently inhibited the phosphorylation of RNAPII at serine 2 residue (i.e. p‐RNAPII^Ser2^), which is the hallmark of CDK9 activity (Natoni *et al*., 2011). As shown in Fig. 4A, treatment of HCT 116 and HT29 cells with CDKI‐73 for 1 h reduced the level of p‐RNAPII^Ser2^ at 0.25 μm concentration and abolished at 1 μm. The level of phosphorylated RNAPII at serine 5 residue (i.e. p‐RNAPII^Ser5^) was also reduced, but to a much lesser extent. As we previously reported that CDKI‐73 also inhibits the phosphorylation of eIF4E (Lam *et al*., 2014), we investigated the proteins involved in the Mnk‐eIF4E axis and found they were not affected after 1‐h treatment. All these confirm that CDK9 is the primary target of CDKI‐73.

To investigate the downstream effect of pharmacological inhibition of CDK9 by CDKI‐73, we extended the treatment period to 24 h in both HCT 116 and HT29 cells. CDKI‐73 0.25 μm resulted in the robust reduction of p‐RNAPII^Ser2^ (Fig. 4B). At 0.25 μm concentration, CDKI‐73 was capable of reducing the expression of Mnk1 and thus blocking the Mnk‐mediated eIF4E phosphorylation at serine 209 (p‐eIF4E^Ser209^) and reducing phosphorylation of ribosomal protein S6 (p‐S6^Ser240/244^) (Fig. 4A,E, top and bottom). From Fig. 4F, we can see that the effect of CDKI‐73 on Mnk1 was at the transcription level, as the MNK1 mRNA was significantly reduced after treatment with 0.25 μm. No changes in the levels of RNAPII or eIF4E proteins were detected. These observations confirmed that CDKI‐73‐targeted CDK9‐mediated transcription of proteins was involved in the translational regulation in HCT 116 and HT29 cells.

We further investigated the effect of CDKI‐73 on the MAPK and mTOR pathways. The activity of Mnk1 is regulated by p38 mitogen‐activated protein kinase (p38 MAPK) and extracellular signal‐regulated kinases (Erks) through phosphorylation at Thr209/214 (Scheper *et al*., 2001; Waskiewicz *et al*., 1997). Western blot analysis of HCT 116 and HT29 cells after 24 h exposure to CDKI‐73 showed minimal effect on the Erk and its phosphorylation (Fig. 4C). The p38 MAPK is activated by mitogen‐activated protein kinase MKK 3/6 through phosphorylation at Thr180 and Tyr182 residues (p‐p38^Thr180/Tyr182^). Again, CDKI‐73 did not affect the expression levels of p38 or p‐p38^Thr180/Tyr182^. However, the phosphorylation of eIF4E‐binding protein 1 (4E‐BP1), i.e. p‐4E‐BP1^Thr70^, was reduced by 0.25 μm CDKI‐73 and decreased levels of 4E‐BP1 and ribosomal protein S6 (rpS6) proteins were also observed. The mRNA level of 4E‐BP1 was also reduced but this was not statistically significant (Fig. 4F).

Recently, we demonstrated that CDKI‐73 causes apoptosis through reduction of Mcl‐1 and c‐Myc in MLL‐AML cell lines (Li *et al*., 2015). Mcl‐1 and c‐Myc are known to be regulated by CDK9‐driven transcription and eIF4E‐mediated translation (Hou *et al*., 2012; Wang and Fischer, 2008). As shown in Fig. 4D, the expressions of Mcl‐1 and c‐Myc were inhibited in HCT 116 cells after treatment with 0.25 μm CDKI‐73. The same treatment also suppressed the level of cyclin D1 protein. The induction of cleaved caspase‐3 and ‐7 confirmed the occurrence of apoptosis. Consistent with our previous studies with other CDK9 inhibitors (Liu *et al*., 2012; Wang *et al*., 2010), the treatment with increasing concentrations of CDKI‐73 led to progressive increases in p53 in HCT 116 cells, accompanied by reductions in MDM2 protein, a short‐lived protein responsible for the ubiquitination of p53 (Fig. 4D). However, we could not detect expression of wild‐type p53 in HT29 cells, as it is a p53‐mutated cell line (Ahmed *et al*., 2013).

### 3.5 Knockdown assay confirms the CDK9‐targeted mechanism of CDKI‐73 and involvement of CDK9 in eIF4E signalling

**Figure 5:**
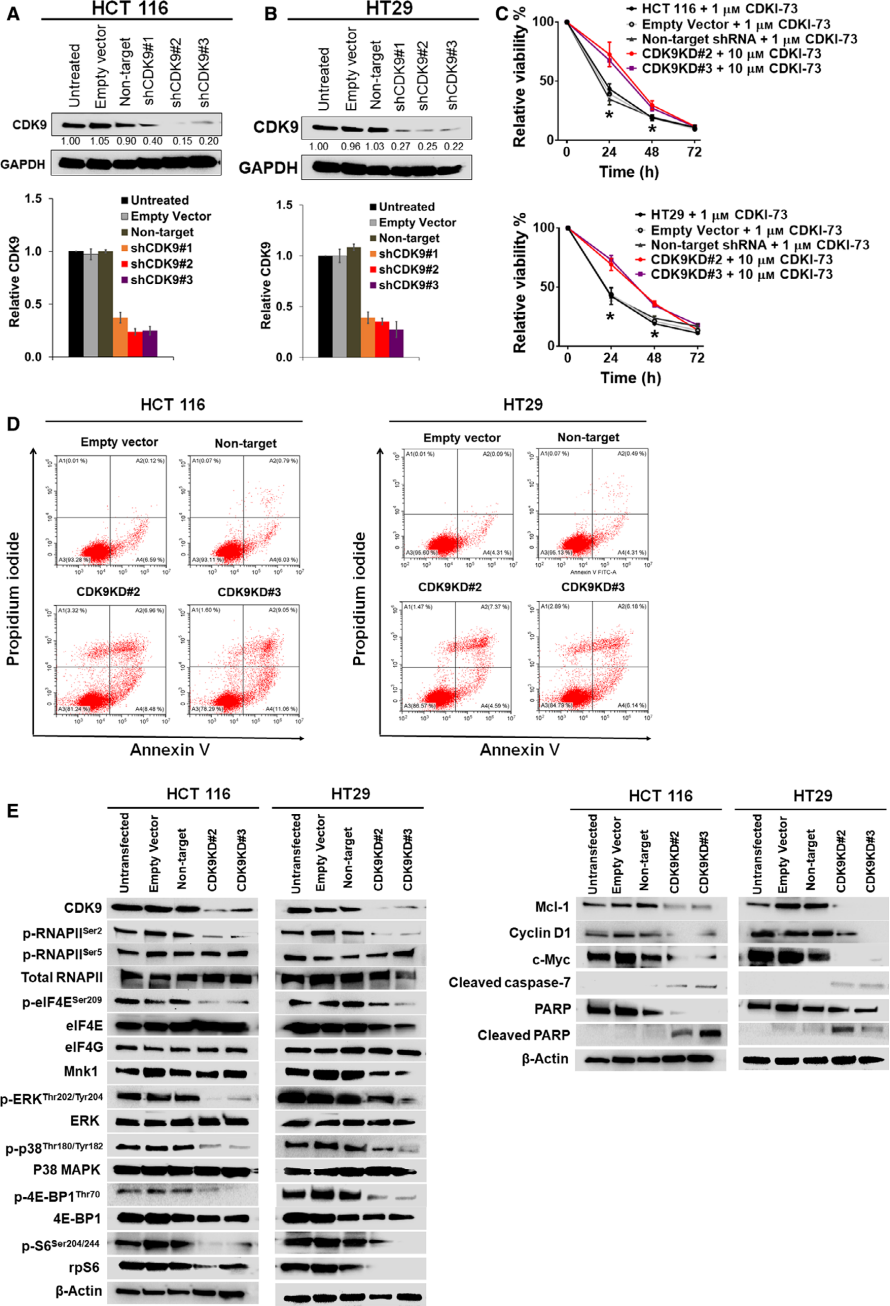
Effects of CDK9 knockdown in CRC cells. (A) Comparison of CDK9 expression in wild‐type and knockdown HCT 116 cells at protein level (top) and relative mRNA level (bottom) of CDK9 in shRNA‐mediated CDK9KD HCT 116 cells. (B) Comparison of CDK9 expression in wild‐type and knockdown HT29 cells at protein level (top) and at mRNA level (bottom). CDK9 expression levels of the empty vector and non‐target shRNA controls are also shown. Western blot showing CDK9 expression in CDK9KD HCT116 cells. (C) Viability of CDK9KD HCT 116 and wild‐type HCT 116 (HCT 116; top and HT29; bottom) cells after treatment with indicated concentration of CDKI‐73 over a period of 0–72 h. Data shown as mean ± SD, *n* = 3, *P <* *0.05, unpaired *t*‐test. (D) Apoptosis analysis in CDK9KD cells. Empty vector and non‐target shRNA controls are also shown. Representative figures are shown from at least two independently repeated experiments. (E) Western blot analysis of HCT 116 and HT29 cells transfected with CDK9 shRNA. Untransfected cells, cells transfected with empty vector, and non‐target shRNA were used as controls. Effects of CDK9KD on Mnk/eIF4E‐mediated translational pathway of these cells were determined using the indicated antibodies. Proteins involved in the apoptotic pathways such as Mcl‐1, cyclin D1, c‐myc, cleaved caspase‐7 and cleaved PARP were also examined. β‐Actin was used as an internal loading control.

shRNA‐mediated knockdown assay was performed to confirm the CDK9‐targeted mechanism of CDKI‐73. Among three CDK9‐targeted shRNA clones, about 80% knockdown of CDK9 was achieved in HCT 116 and HT29 cells using shCDK9#2 and shCDK9#3 compared with untreated and shRNA controls (empty vector and non‐target) (Fig. 5A,B). Notably, CDK9 knockdown conferred resistance to CDKI‐73 in HCT 116 and HT29 cells (by more than 100‐fold and 10‐fold, respectively) for at least 24 h treatment (24 h, IC_50_ > 10 μm; Table S1). Treatment of CDK9 knockdown (CDK9KD#2 and CDK9KD#3) HCT 116 and HT29 cells with 10 μm CDKI‐73 over a period of 0–72 h revealed that CDKI‐73 was significantly less potent against those cells (*P *<* *0.05) compared with 1 μm CDKI‐73‐treated wild‐type HCT 116 (Fig. 5C, top) and HT29 (Fig. 5C, bottom) cells. Cells transfected with empty vector and non‐target shRNA also showed a similar level of sensitivity to the wild‐type cells. The resistance to CDKI‐73 treatment in CDK9KD cells compared with wild‐type cells provided evidence of its CDK9‐targeted mechanism. Moreover, knockdown of CDK9 caused apoptosis in HCT 116 and HT29 cells, whereas empty vector and non‐targeted shRNA did not cause any apoptosis (Fig. 5D). To understand whether the reduction of eIF4E‐mediated translation by CDKI‐73 was driven by its inhibition of CDK9, we investigated the effect of CDK9 knockdown on Mnk‐eIF4E pathways in CDK9KD HCT 116 and CDK9KD HT29 cells compared with untransfected cells and shRNA controls (empty vector and non‐target). The protein levels of CDK9, p‐RNAPII^Ser2^ and p‐eIF4E^Ser209^ were reduced in the CDK9KD cells (Fig. 5E). Consistent with our previous finding in A2780 ovarian cancer cell line, CDK9 knockdown did not affect the Mnk1 protein in HCT 116 cells. However, Mnk1 was slightly reduced in CDK9KD HT29 cells.

The CDK9KD cells also showed a reduction in p‐Erk^Thr202/Tyr204^ without affecting the Erk protein (Fig. 5E), suggesting a role of CDK9 on eIF4E‐Mnk‐mediated translation. The expression of 4E‐BP1 and rpS6, and their phosphorylation were influenced by the knockdown CDK9. This indicated a downstream effect of CDK9 knockdown on the translation pathway. However, only the phosphorylation of p38 (p‐p38^Thr202/Tyr204^) was reduced; the total p38 remained unaffected. CDK9 knockdown also had an effect on the expression levels of c‐myc, cyclin D1, cleaved caspase‐7 and PARP (Fig. 5E), correlating with the apoptotic effect shown in the CDK9KD HCT 116 and CDK9KD HT29 cells (Fig. 5D).

### 3.6 CDKI‐73 suppresses the growth of colorectal tumour by targeting CDK9 *in vivo*

**Figure 6:**
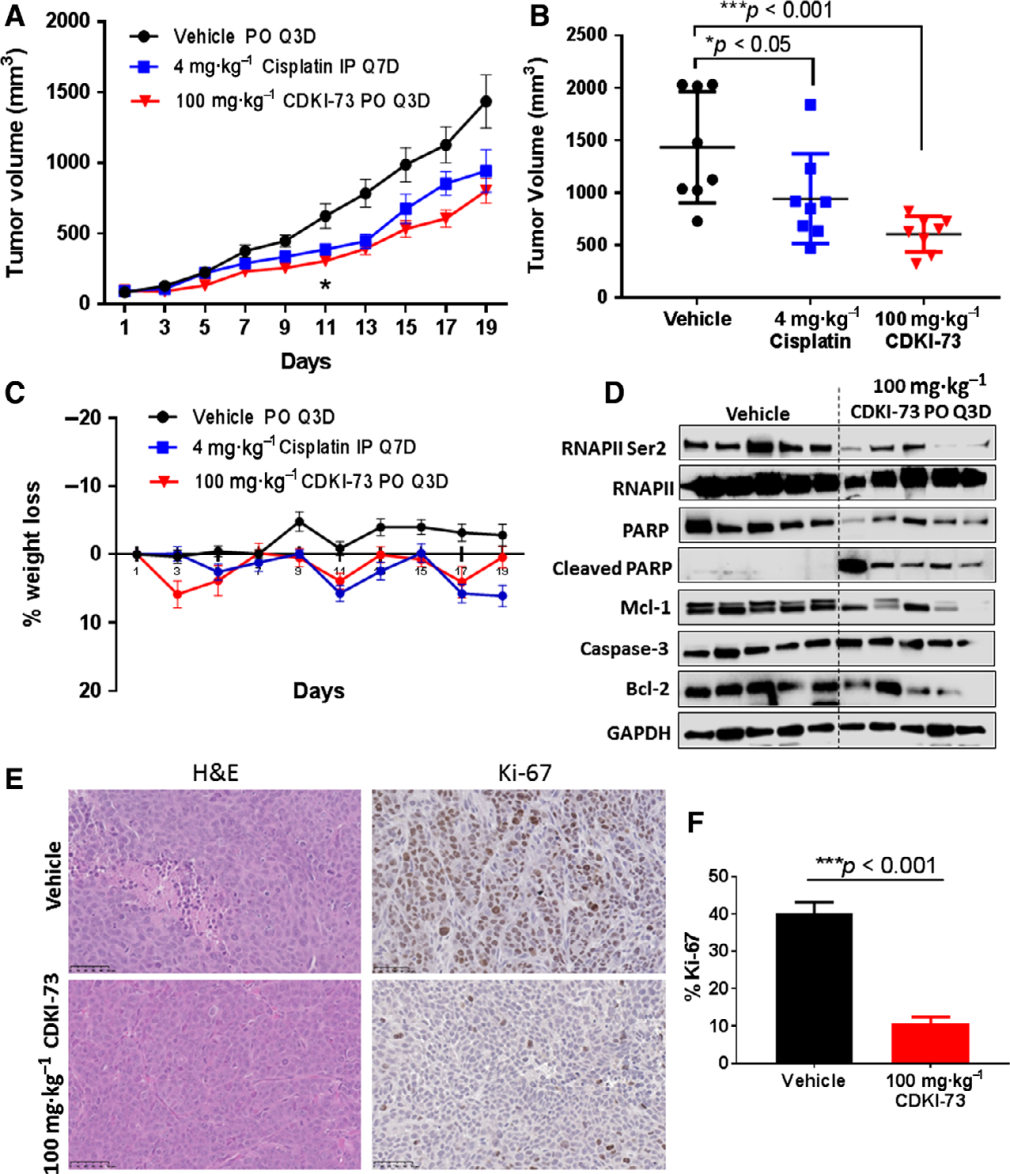
*In vivo* anti‐tumour efficacy of CDKI‐73 in HCT 116 xenograft model. Groups of eight animals were administered vehicle, cisplatin (4 mg·kg^−1^, IP, Q7D) or CDKI‐73 (100 mg·kg^−1^, PO, Q3D). (A) Graph showing tumour volume at different days in group of mice receiving specific treatment (mean ± SEM). *P <* *0.05 (one‐way ANOVA). (B) Scatter plot showing comparison and individual tumour volume among the three groups on day 19 (horizontal line indicates the means and vertical lines indicate standard deviations). One‐way ANOVA was used to determine statistical significance. (C) Graph showing body weight loss in different groups. (D) Western blot analysis of tumour tissues collected from the xenograft studies. GAPDH was used as a loading control. (E) IHC analysis for proliferative activity (Ki67) in HCT 116 xenograft tumour tissues (scale bar: 50 μm). (F) Five fields were randomly selected from each tissue (from five mice in each group) for quantitation of proliferative index. Data presented as mean ± SEM. Unpaired *t*‐test was used to determine the *P*‐values.

The greater advantage of CDKI‐73 over the clinical experimental CDK9 inhibitors, i.e. flavopiridol and dinaciclib, is its excellent drug property with high oral bioavailability, which make it possible to be orally administrable to patients. We confirmed the therapeutic efficacy of CDKI‐73 in an HCT 116 xenograft model. As shown in Fig. 6A, HCT 116 tumour‐bearing animals treated with 100 mg·kg^−1^ CDKI‐73 orally once every 3 days caused a significant tumour growth inhibition (*P *<* *0.05) from day 11 onwards when compared with the vehicle group. Cisplatin was also efficacious compared with the vehicle. CDKI‐73 showed significant inhibition of tumour growth when compared with cisplatin on day 17 (*P *≤* *0.05). Further treatment with CDKI‐73 resulted in marked reduction in the tumour volumes by day 19 (Fig. 6B, *P* < 0.001). There was no significant bodyweight loss or other clinical signs of toxicity in CDKI‐73‐treated mice (Fig. 6C).

Furthermore, we have investigated the *in vivo* mechanism of tumour growth inhibition by utilising western blot and IHC analysis of the tumours collected from the xenografted animal treated with CDKI‐73 or vehicle (*n* = 5). The treatment with CDKI‐73 caused robust reduction of p‐RNAPII^Ser2^, confirming inhibition of CDK9 kinase activity. In line with our *in vitro* findings, CDKI‐73 also reduced the level of Mcl‐1 and Bcl‐2, which was accompanied by induction of apoptosis indicated by cleavage of PARP when compared with the vehicle‐treated tumours (Fig. 6D). IHC analysis of these tumour tissues showed that CDKI‐73 markedly reduces the proliferation, as indicated by a significant decreased in the level of Ki‐67‐positive cells (Fig. 6E,F, *P* < 0.001).

## 4 Discussion

CDK9 is highly expressed in CRC (Uhlen *et al*., 2015). Promising *in vitro* targeting profile of CDKI‐73 against a wide range of cancer cell lines and its low toxicity towards normal cells led to this current study. Consistent with our previous findings (Lam *et al*., 2014; Walsby *et al*., 2014), CDKI‐73 manifested potent anti‐proliferative effects against all HCCL tested, irrespective of the cellular characteristics, e.g. histopathology, differentiation and clinical stage of disease. As the IC_50_ value is correlated to cell doubling time, time‐dependent sensitivity to CDKI‐73 was consistent with the previously reported doubling time of the HCCL tested (Bazzocco *et al*., 2015; Dahlmann, 2013). Having the shortest doubling time compared with the other cell lines, HCT 116 was the most sensitive to CDKI‐73 treatment from 24 h and onwards. Cellular CDK9 targeting specificity of CDKI‐73 was demonstrated by comparing its effect in wild‐type and CDK9KD (HCT 116 and HT29) cells. As CDK9KD cells already have a reduced level of CDK9 activity, accompanied by a reduced level of anti‐apoptotic proteins (Mcl‐1, c‐Myc and cyclin D1; Fig. 5E), they were less sensitive than the cells expressing CDK9 (Fig. 5C, Table S1). CDKI‐73 reduced the phosphorylation of RNAPII (i.e. p‐RNAPII^Ser2^, Fig. 4A), supporting the notion that CDKI‐73 primarily targets CDK9.

CDKI‐73 also reduced cellular Mnk activity (Fig. 4B) and thus suppressed the eIF4E‐mediated translation. As p‐eIF4E has been found to be elevated in CRC (Diab *et al*., 2014), this additional inhibitory effect might contribute to the rapid induction of apoptosis in HCT 116 cells. The role of CDK9 in the Mnk‐eIF4E axis was confirmed by the simultaneous reduction of p‐RNAPII^Ser2^ and the Mnk‐activating kinases (i.e. p‐Erk^Thr202/Tyr204^) in the CDK9KD cells (Fig. 5E). Although Erk phosphorylation was reduced in CDK9KD cells, it was barely affected by pharmacological inhibition via CDKI‐73 (Figs 4C and 5E). Although the underlying cause remains to be elucidated, a previous study has implicated Erk in assembly of P‐TEFb by stabilising CDK9 (Fujita *et al*., 2008). The reduced level of p‐Erk^Thr202/Tyr204^ could be due to the down‐regulation of CDK9‐associated genomic network in the CDK9KD cells. Clearly, there is a discrepancy between pharmacological inhibition and gene silencing. CDKI‐73 blocks the function of CDK9 but the protein is still present, whereas shRNA removed CDK9 mRNA and its protein from the cell, consequently affecting the network of genes and proteins that are associated with CDK9, including the Mnk‐eIF4E axis.

Cell cycle analysis of HCT 116 and HT29 cells treated with CDKI‐73 showed substantial cell death (i.e. sub‐G1 cell populations, Fig. 2A), which was confirmed by the detection of apoptotic cells (Fig. 2B). CDKI‐73 retained the ability to induce apoptosis in the presence of a pan‐caspase inhibitor Z‐VAD‐FMK, suggesting it can cause caspase‐independent apoptosis. One plausible explanation is that CDKI‐73 inhibits the transcription of key genes for mitochondrial function, such as Mcl‐1, which plays an essential role in the maintenance of membrane stability (Gojo *et al*., 2002; Minagawa *et al*., 2005).

In addition to their oncogenic properties, Bcl‐2, cyclin D1 and Mcl‐1 play a role in mitochondrial function and stability (Harris and Thompson, 2000; Minagawa *et al*., 2005; Sakamaki *et al*., 2006). Our previous studies have shown that CDKI‐73 induced apoptosis by targeting short‐lived pro‐survival mRNA, such as Mcl‐1, XIAP and Bcl‐2 in cancer cells (Lam *et al*., 2014; Walsby *et al*., 2014). Thus, transcriptional repression of these factors leads to depolarisation of mitochondrial membrane, and results in the release of apoptogenic factors including apoptosis‐inducing factor and endonuclease G triggering caspase‐independent apoptosis (Kroemer *et al*., 2007). Consistent with a previous report that flavopiridol caused early mitochondria damage and induced caspase‐independent apoptosis through the transcription inhibition of Mcl‐1 (Hussain *et al*., 2008), CDKI‐73 inhibited Mcl‐1 at both mRNA and protein levels (Fig. 3A,B). However, the reduction of cyclin D1 at the mRNA level was not as prominent as the reduction at the protein level. The combined effect of down‐regulation of transcription and eIF4E‐mediated translation caused by CDK9 inhibition might have led to such striking effect at the cyclin D1 protein level. Furthermore, in line with our previous findings (Lam *et al*., 2014; Liu *et al*., 2012), CDKI‐73 elevated the level of p53 with a concomitant reduction in MDM2 (Fig. 4D). Interestingly, CDKI‐73 also caused apoptosis through simultaneous inhibition of Mcl‐1, c‐Myc and cyclin D1 in the p53‐mutated HT29 cells (Fig. 4D).

Oral administration of CDKI‐73 demonstrated an excellent anti‐tumour efficacy in an HCT 116 xenograft model (Fig. 6A,B). Although they appeared to share a similar mechanism of action *in vitro*, CDKI‐73 has an advantage of better pharmacokinetics and higher oral bioavailability than flavopiridol. Moreover, the dose‐limiting toxicity due to lack of selectivity and prolonged CDK9 inhibition narrowed the therapeutic index of flavopiridol (Dey *et al*., 2017; Lanasa *et al*., 2015), whereas CDKI‐73 has shown a remarkably reduction of toxicity in non‐cancerous cells (Walsby *et al*., 2014). Western blot analysis of the tumours collected from xenografted mice treated with CDKI‐73 confirmed its inhibition of CDK9 *in vivo* (Fig. 5E). The treatment resulted in the reduction of known CDK9 targets, i.e. Bcl‐2 and Mcl‐1 in tumour tissues. In addition to that, a significantly lower Ki‐67 proliferative index (Fig. 6F, *P* < 0.001) predicted increased survival in CDKI‐73‐treated mice (Oshima *et al*., 2005). These findings emphasise the therapeutic potential of CDKI‐73 for treatment of CRC.

## 5 Conclusions

We have identified CDKI‐73 as a highly potent anti‐proliferative agent against HCCL. CDKI‐73 effectively induced cancer cell death and displayed a robust CDK9‐targeted mechanism of action both *in vitro* and *in vivo*. It down‐regulates multiple anti‐apoptotic factors and inhibits tumour growth of the HCT 116 xenograft by oral administration. The high potency and low toxicity make CDKI‐73 a highly attractive candidate for development in the clinic.

## Conflict of interest

SW has ownership interest (including patents) and is a consultant/board member of Changzhou LeSun Pharmaceuticals Ltd. The other authors have no conflicts of interest to declare.

## Author contributions

SW conceived and designed the project. MHR, FL, LZ, JA, CP, NAL, CR and MKO conducted the experiments and data analysis. MHR, SW, FL, TT, MY, RWM, NAL and CR wrote and/or revised the manuscript.

## Supporting information


**Table S1.** Time‐course anti‐proliferative effect of CDKI‐73 on wild‐type and CDK9 knockdown HCT 116 and HT29 cells determined by MTT assays.
**Fig. S1.** Cell cycle analysis in CDK9 knockdown cells.
