# Supplementary material for: Targeting CDK9 for treatment of colorectal cancer
Source: Mol Oncol. 2019 Aug 21;13(10):2178–93. doi: 10.1002/1878-0261.12559 (PMC6763784; doi:10.1002/1878-0261.12559)
Supplement: Supplementary file 1 — Table S1. Time‐course anti‐proliferative effect of CDKI‐73 on wild‐type and CDK9 knockdown HCT 116 and HT29 cells determined by MTT assays. Fig. S1. Cell cycle analysis in CDK9 knockdown cells. [file MOL2-13-2178-s001.docx]

**Supporting information**

**Supplementary Table S1.** Time-course anti-proliferative effect of CDKI-73 on wild-type and knockdown HCT 116 and HT29 cells determined by MTT assays

| **HCCL** | **Compound** | **IC_50_ (μM) ± SD^*^** | | |
| --- | --- | --- | --- | --- |
|  |  | **24 h** | **48 h** | **72 h** |
| HCT 116 | CDKI-73 | 0.081 ± 0.019 | 0.063 ± 0.006 | 0.017 ± 0.007 |
|  | Flavopiridol | 0.068 ± 0.007 | 0.041 ± 0.004 | 0.027 ± 0.014 |
| HCT 116 CDK9KD#2 | CDKI-73 | > 10 | 0.039 ± 0.008 | 0.037 ± 0.013 |
| HCT 116 CDK9KD#2 | CDKI-73 | > 10 | 0.039 ± 0.012 | 0.032 ± 0.009 |
| HT-29 | CDKI-73 | 0.903 ± 0.014 | 0.068 ± 0.001 | 0.039 ± 0.013 |
|  | Flavopiridol | 0.531 ± 0.059 | 0.035 ± 0.011 | 0.027 ± 0.009 |
| HT-29 CDK9KD#2 | CDKI-73 | >10 | 0.084 ± 0.015 | 0.032 ± 0.008 |
| HT-29 CDK9KD#3 | CDKI-73 | >10 | 0.132 ± 0.028 | 0.039 ± 0.011 |

*Data given are the mean ± SD derived from at least three replicates.


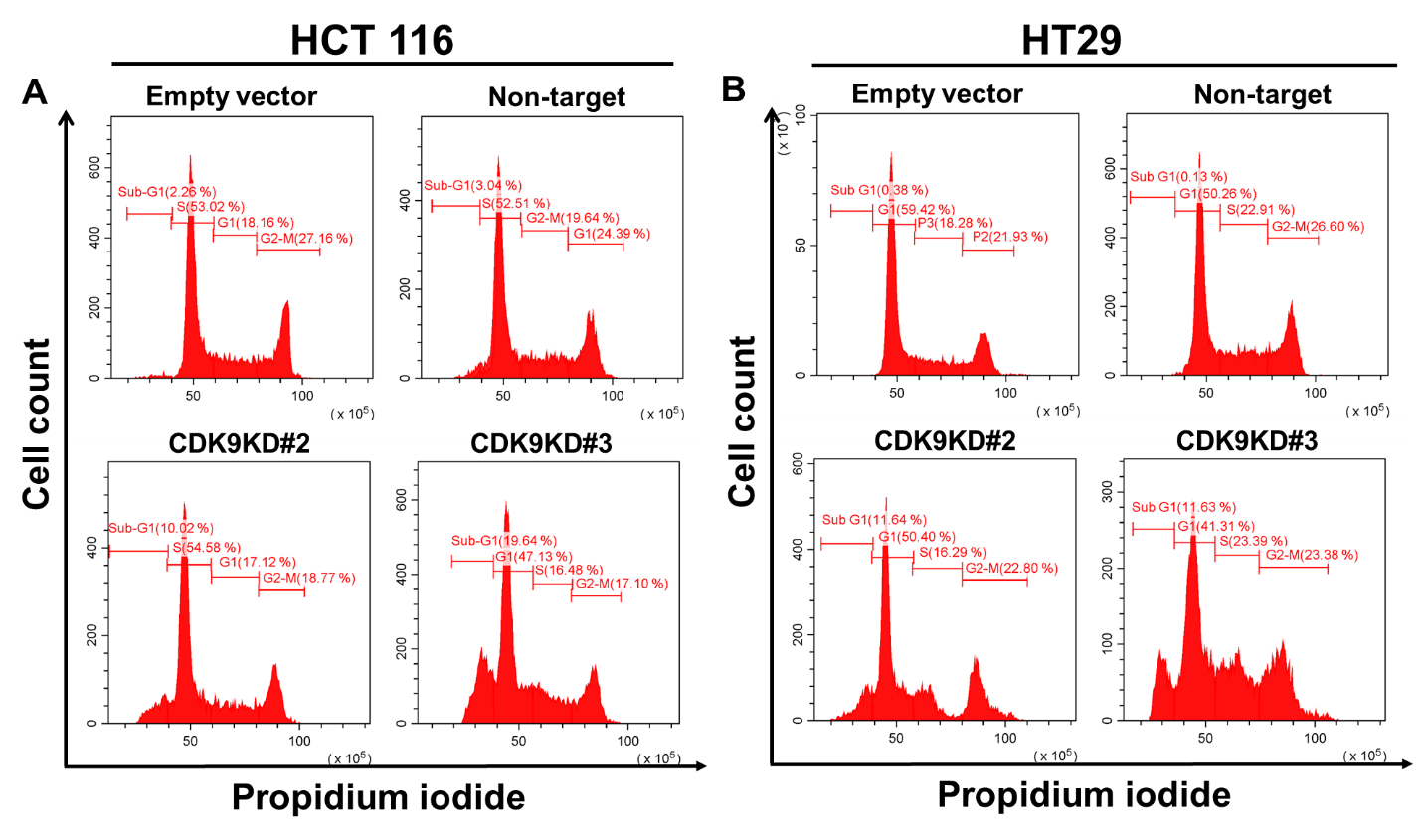
**Supplementary Fig. S1:** Cell cycle in CDK9 knockdown cells. (A) HCT 116 and (B) HT29 cells. Empty vector and non-target shRNA controls are also shown. Representative figures are shown from at least two independently repeated experiments.
